# Spinal afferent neurons: emerging regulators of energy balance and metabolism

**DOI:** 10.3389/fnmol.2024.1479876

**Published:** 2024-11-08

**Authors:** Mohammad Jarrah, Dana Tasabehji, Aviva Fraer, Mohamad Mokadem

**Affiliations:** ^1^Department of Internal Medicine, Roy J. and Lucille A. Carver College of Medicine, University of Iowa, Iowa City, IA, United States; ^2^Iowa Neuroscience Institute, Roy J. and Lucille A. Carver College of Medicine, University of Iowa, Iowa City, IA, United States; ^3^Fraternal Orders of Eagles Diabetes Research Center, University of Iowa, Iowa City, IA, United States; ^4^Obesity Research and Education Initiative, University of Iowa, Iowa City, IA, United States; ^5^Veterans Affairs Health Care System, Iowa City, IA, United States

**Keywords:** spinal afferent neurons, energy homeostasis, gut-brain axis, food intake regulation, metabolic regulation

## Abstract

Recent advancements in neurophysiology have challenged the long-held paradigm that vagal afferents serve as the primary conduits for physiological signals governing food intake and energy expenditure. An expanding body of evidence now illuminates the critical role of spinal afferent neurons in these processes, necessitating a reevaluation of our understanding of energy homeostasis regulation. This comprehensive review synthesizes cutting-edge research elucidating the multifaceted functions of spinal afferent neurons in maintaining metabolic equilibrium. Once predominantly associated with nociception and pathological states, these neurons are now recognized as integral components in the intricate network regulating feeding behavior, nutrient sensing, and energy balance. We explore the role of spinal afferents in food intake and how these neurons contribute to satiation signaling and meal termination through complex gut-brain axis pathways. The review also delves into the developing evidence that spinal afferents play a crucial role in energy expenditure regulation. We explore the ability of these neuronal fibers to carry signals that can modulate feeding behavior as well as adaptive thermogenesis in adipose tissue influencing basal metabolic rate, and thereby contributing to overall energy balance. This comprehensive analysis not only challenges existing paradigms but also opens new avenues for therapeutic interventions suggesting potential targets for treating metabolic disorders. In conclusion, this review highlights the need for a shift in our understanding of energy homeostasis, positioning spinal afferent neurons as key players in the intricate web of metabolic regulation.

## Introduction

Modern theories and frameworks addressing the biological determinants of energy homeostasis hypothesize that a multitude of external (environmental) and internal (endogenous) stimuli are processed within the central nervous system (CNS). This integrative mechanism results in synchronized alterations in both caloric consumption and energy utilization. These adjustments are mediated through a complex interplay of behavioral responses, autonomic nervous system activities, and endocrine system functions, collectively acting as effector pathways (Berthoud, 2002; Schwartz et al., 2017). Increasing evidence highlights the pivotal role of central and peripheral nervous systems in regulating energy balance, revealing how environmental and endogenous factors can modulate energy crosstalk between organs, especially along the gut-brain axis (Yengo et al., 2018; Kweon et al., 2022; Morys et al., 2024). This would allow us to further deepen our understanding of the neural mechanisms underlying obesity to advance and diversify treatment strategies. A key question is whether the primary defect lies in peripheral tissues (like adipose tissue, liver, and gut) or CNS. The hypothalamus, a crucial CNS region, has long been recognized as a key regulator of feeding behavior and metabolism where it senses the body’s energy states in response to neuro-hormonal signals and the presence of nutrients in the blood (Kim, 2022). The brain does not function in isolation, and it relies on both external and internal clues to make decisions (Roh et al., 2016; Kim, 2022). These decisions are strongly influenced by interoception, which is the collection of senses providing information about the body’s internal state. Interoception involves both humoral and neural communication between the periphery and the brain, and is influenced by behavioral, autonomic, and endocrine pathways (Münzberg et al., 2023). The CNS intricately links with peripheral organs, including the gastrointestinal (GI) tract and the peripheral nervous system, via the autonomic nervous system (ANS). This sophisticated network orchestrates the regulation of food intake and energy expenditure (Kim, 2022). Vagal afferent neurons have traditionally been the focus of this gut-brain communication (Forsythe et al., 2014; Bonaz et al., 2018). However, recent research suggests a noteworthy metabolic regulatory role of spinal afferent neurons along the gut-brain axis. The spinal sensory pathways, encompassing the dorsal root ganglia (DRG), extend beyond the vagally innervated viscera to also encompass adipose tissue, skeletal muscle, and skin, thereby supplying the brain with extensive metabolic information (Mishra and Townsend, 2023). Although spinal afferents are well-documented in the context of pain related to intestinal diseases such as irritable bowel syndrome, inflammatory bowel disease, and motility disorders, there is limited information about their role in regulating energy metabolism (Smith-Edwards et al., 2019; Abdullah et al., 2020; Gershon and Margolis, 2021). This opens the door to further the exploration of the actual functions these neurons serve.

Understanding the complex interconnections between the CNS, spinal afferent neurons, and peripheral metabolic organs is essential for comprehending the regulation of energy expenditure, thermogenesis, and physical activity (Kim, 2022). These findings underscore the critical role of spinal afferent neurons within the regulatory networks governing energy homeostasis paving the way for potential therapeutic strategies targeting metabolic disorders and associated pathologies. The primary aim of this review is to shine light on the potential contribution of spinal sensory pathways to metabolism and explore available data on their facilitating role in regulation of food intake and energy balance.

## Neuroanatomical insights of energy homeostasis mediated by sensory neurons

The hypothalamus, which regulates multiple homeostatic functions- including energy balance-, establishes a crucial connection with the GI tract through vagal and spinal neuronal fibers, thereby influencing energy intake and metabolic rate to maintain body weight homeostasis (Schwartz et al., 2000; Galgani and Ravussin, 2008). The parasympathetic nervous system relays information concerning food ingestion to the CNS, thereby facilitating satiety by modulating gastric motility and emptying processes (Browning and Travagli, 2014). Conversely, the sympathetic nervous system plays a pivotal role in regulating energy expenditure; it innervates the mesenteric gut and serves in regulating gut motility, digestive secretions as well as lipid turnover and thermogenesis within (WAT) (Bowers et al., 2004; Rao and Gershon, 2016). Peripheral axons of visceral DRG afferent fibers travel along with autonomic efferent in thoracic, lumbar splanchnic sympathetic, and pelvic parasympathetic nerves giving collaterals to neurons in prevertebral ganglia such as celiac and superior mesenteric ganglia (Jänig, 2022). Sensory information from the GI tract can be mediated to the spinal cord by enteric neurons projecting to prevertebral ganglia and to the spinal cord (Münzberg et al., 2023). Different regions of the GI tract, liver, pancreas, adipose tissues, skin and skeletal muscles are innervated by DRG neurons, with specific segments of the thoracic and lumbosacral spine providing neural input to these areas (Münzberg et al., 2023).

The gut-brain axis has been strongly implicated in control of food intake and regulation of energy homeostasis through a complex interconnected neuro-hormonal network (Clemmensen et al., 2017). The “hormonal” branch of this network focuses on gut-released peptides- such as cholecystokinin, glucagon-like peptide-1 (GLP-1), gastric inhibitory peptide, Protein YY- which can act as incretins to prompt post-ingestion insulin secretion and/or regulators of food intake and energy expenditure to prompt weight balance (Hintze et al., 2017). The “neuronal” branch of this energy crosstalk pathway was previously focusing on exploring the role of vagal afferents in this communication where vagal afferents were thought to mainly mediate signals for normal physiological regulation of digestion, nutrition, and satiation while spinal afferents mediate signals associated with pathological conditions such as painful overdistention leaving a little room for spinal afferent (Grundy, 2006). Interestingly, it was recently found that subcutaneous WAT and brown adipose tissue (BAT) in mice are exclusively innervated by spinal afferents and not vagal afferents and that this spinal mediated tract plays a key role in regulating adipose tissue mass, lipolysis, and thermogenesis (Münzberg et al., 2023). Spinal afferent neurons, or primary sensory neurons, are integral components of the somatosensory system, responsible for conveying sensory information from peripheral tissues such as the GI tract, liver, and adipose tissue, to the CNS. These neuronally transmitted information are crucial for transmitting signals that regulate appetite and maintain energy homeostasis (Grill and Hayes, 2012; Dunn and Adams, 2014). While most spinal afferents innervate the skin and skeletal muscles, approximately 2% target visceral organs such as the GI tract, liver, and pancreas (Jänig, 2022). Their cell bodies reside in the DRG, with peripheral axons extending to various tissues and organs, including the GI tract, where they detect mechanical and chemical stimuli related to food intake (Chen et al., 2015). A significant part of the published research on DRG and afferent neurons focuses on their roles in pain mechanisms, somatosensory physiology, peripheral nerve function, and spinal cord injury (Smith-Edwards et al., 2019; Abdullah et al., 2020; Gershon and Margolis, 2021). On the other hand, there are fewer studies that systematically explore the role of spinal sensory neurons in energy regulation. Among those, a recent study highlights the role of spinal afferent fibers and sympathetic efferent fibers in lipolysis, short-term food intake, and thermogenesis, and states that this communication is also crucial for the beneficial metabolic effects observed in bariatric surgeries such as Roux-en-Y gastric bypass in mice, as splanchnic denervation was shown to negate these well-documented benefits (Ye et al., 2020). Understanding the mechanisms by which these neurons contribute to energy balance is essential for gaining insights into the neurobiology of food intake regulation and developing potential therapeutic targets for managing eating-related disorders and metabolic diseases. Sensory afferent neurons, particularly those located in the DRG, play a key role in transmitting signals related to various stimuli, including nutrients. These neurons help regulate metabolic functions by sensing nutrients like glucose in the intestine and relaying this information to the brain. Spinal afferents are stimulated by glucose sensors in the intestine and hepatic portal vein, and they transfer this signaling to the brain (Goldstein et al., 2021). Disruptions in this nutrient-sensing pathway, often due to conditions like inflammation or diabetic neuropathy, can impair gut-brain communication, leading to disordered eating behavior, as well as disrupted energy and glucose metabolism (Yamada and Monai, 2023). Proper functioning of these pathways is essential for maintaining energy balance and metabolic homeostasis. Furthermore, sensory afferent neurons from visceral and inguinal WAT to the brain have been postulated by many, with a suggested role of communicating energy signals from adipose tissue to energy-regulating areas in the CNS. There are several pieces of evidence demonstrating that these fat-originating spinal sensory neurons can modulate directly and indirectly the activity of sympathetic input to BAT; regulating thermogenesis and overall energy homeostasis (Song et al., 2009; Wang et al., 2022; Mishra and Townsend, 2023). Others have shown that sensory neurons can affect exercise motivation and performance by transporting microbiota signaling from the gut to the midbrain (Dohnalová et al., 2022).

## Spinal afferent neurons and food intake regulation

**Figure 1:**
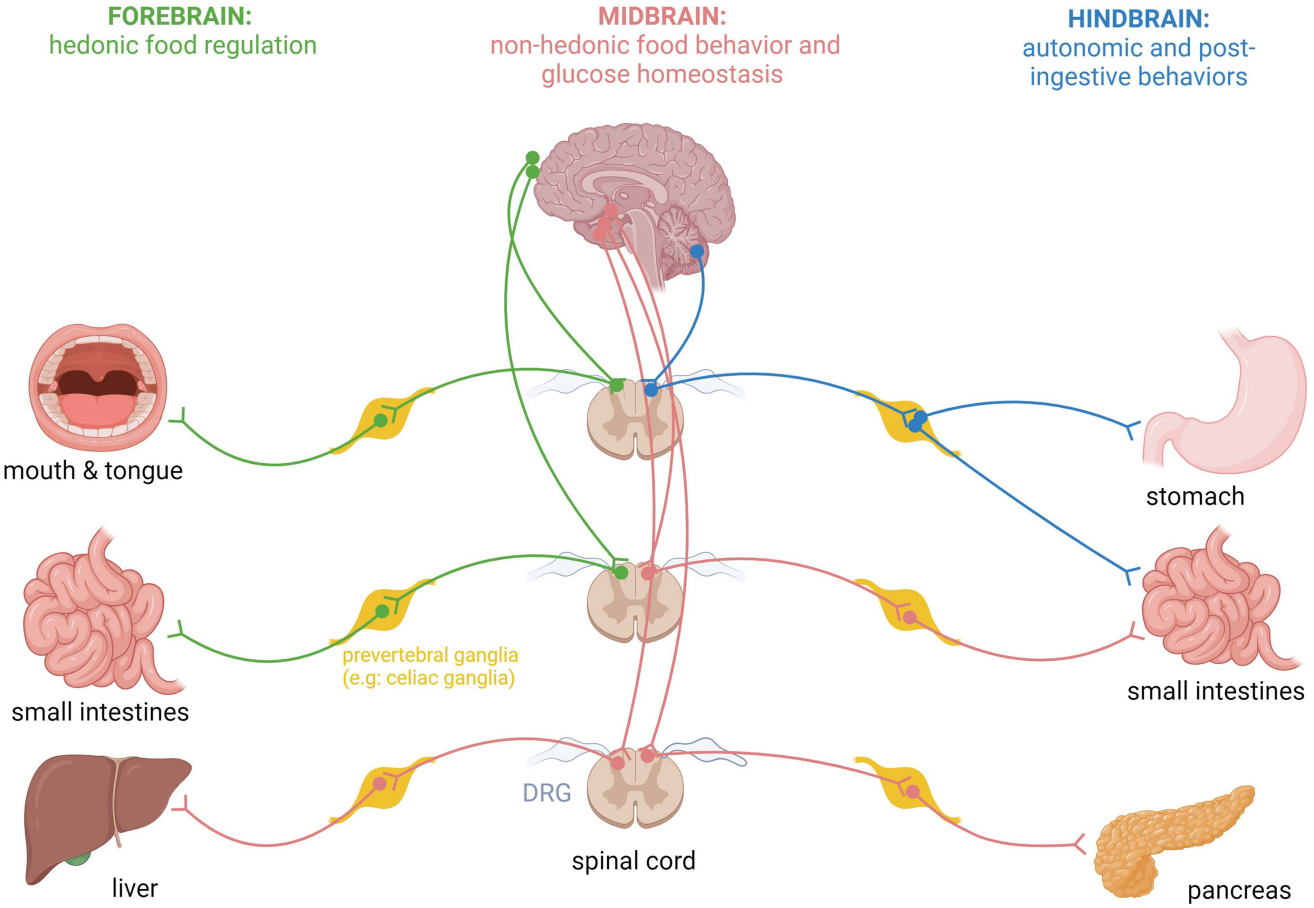
Schematic representation of specific spinal sensory fiber projections from internal organs to forebrain, midbrain, and hindbrain areas for regulation of specific feeding behaviors and glucose homeostasis.

It was traditionally accepted that vagal afferents are the main neuronal fibers responsible for transmitting signals related to normal physiological functions. In contrast, spinal afferents are predominantly associated with pathological states, such as pain induced by excessive distension (Grundy, 2004). This viewpoint has overlooked the possible role of spinal afferents in conveying nutritionally relevant information. Earlier observation in rats revealed that surgical resection of the greater splanchnic nerve abolishes the physiologic compensatory post-ingestion reaction but left total food consumption intact. These findings suggested a role for spinal afferents in the satiation and meal-termination process (Deutsch and Jang Ahn, 1986). One study found that celiac-superior mesenteric ganglionectomy (CSMG) in rats significantly induced reduction in food intake and impaired the sympathoadrenal response to insulin-induced hypoglycemia. Another study demonstrated that CSMG led to disruption of glucose homeostasis by modulating pancreatic beta cell mass and insulin secretion (Imai et al., 2008). Both studies support the involvement of spinal afferents in glucose sensing and food intake regulation. Neurons in the gustatory system, particularly those in the basal forebrain, were shown to respond to food-related stimuli such as odors which can influence feeding behaviors such as meal initiation and satiety (Chen et al., 2015). The study demonstrated that these neurons, when activated by food odors, could prompt feeding even in the absence of actual food consumption, highlighting their role in meal initiation. Additionally, these neurons showed differential activation based on hunger states, indicating their involvement in satiety signaling as well. Conversely, two more comprehensive studies demonstrated that flavor preference learning in rats, facilitated by intragastric or intraduodenal nutrient infusion, is significantly attenuated by celiac/splanchnic mesenteric ganglionectomy, but not by non-selective subdiaphragmatic or selective sensory abdominal vagotomy. These findings suggest a role for splanchnic and spinal afferents in peripheral nutrient sensing (Sclafani and Lucas, 1996; Sclafani et al., 2003). It was also shown that glucose suppresses basomedial hypothalamic Agouti-related peptide (AgRP) neurons (key regulators of energy sensing and food intake) through spinal afferent neurons while fat suppresses their activity via vagal afferent tracts (Goldstein et al., 2021). Furthermore, another study showed that sensing luminal gut osmolarity was robust in glutamatergic vagal neurons but not in DRG neurons, suggesting a segregate role of vagal vs. spinal afferent fibers in differential nutrient sensing (Fajardo et al., 2008). Finally, it was shown- using a high selective DRG de-afferenation technique- that spinal afferent fibers of the stomach play a role in food intake suppression post gastric distention in a pathway that was dependent on GLP-1 signaling. This role was previously attributed solely to the vagus (Zhang et al., 2022). Furthermore, a recent study demonstrated that voltage-gated sodium channel Nav1.8 neurons which are known to be involved in nociception and pain (and present abundantly in spinal afferent neurons) mediate gastric distention signals from the gut as well as nutrient and immune sensing which ultimately regulate diurnal food intake behavior and body weight (Bullich-Vilarrubias et al., 2024). Moreover, foods that are known to contain larger amounts of intragastric nutrients act as positive reinforcers that can activate the brain’s reward system which dictates food selection and increases the desirability of food that is considered beneficial through the use of sensory cues such as sight and smell (Berthoud et al., 2021). These neurons, which are sensitive to ghrelin and leptin, project to specific brain regions within the hypothalamus and hindbrain, such as proopiomelanocortin neurons of the arcuate nucleus, neuropeptide Y (NPY) and AgRP neurons of the paraventricular nucleus of the hypothalamus and ventromedial hypothalamus (VMH) in addition to hindbrain dorsal vagal complex (DVC) and parabrachial nucleus to ultimately regulate feeding behavior (Williams and Elmquist, 2012). Vagal afferent neurons transmit nutrient signaling (specifically glucose) to feeding centers of the brain such as DVC and VMH through sensors in the hepatic portal vein to regulate energy intake indirectly and liver glucose trafficking. However, despite the evidence that DRG neurons enervate the portal vein, no similar effect or role has been shown in spinal afferent neurons (Berthoud et al., 2024). It has been also suggested that palatability does not affect the overall amount of food consumption but the composition itself and repetitive behavior of seeking it, with a likelihood of a direct gut-brain neuronal connection of regulation non-hedonic food intake behavior (de Araujo et al., 2020). The possibility and likelihood of vagal and spinal afferent pathways being part of it is high but not yet well defined. The intricate role of sensory spinal neurons on energy and metabolism is fascinatingly distinct across different aspects of feeding behavior and nutrient sensing. In the forebrain, projections of these neurons seem to shape emotional eating, food preference, and palatability through neuro-gustatory sensory stimuli such as odors and nutrient sensing. Sensory projections to midbrain/hypothalamus are likely involved in modulating homeostatic (non-hedonic) food behavior and glucose homeostasis while brainstem/DVC projections seem more crucial in the regulation of autonomic and post-ingestive behaviors such as satiation (Figure 1).

## Spinal afferent neurons and energy expenditure

Growing evidence also supports the role of spinal sensory neurons in regulating energy expenditure, specifically fat thermogenesis. These neurons receive and process sensory information from the periphery and convey it to the energy regulating centers in the brain to regulate energy expenditure by modulating basal metabolic rate, fat thermogenesis and voluntary physical activity (Grill and Kaplan, 2002; Grill and Hayes, 2012; Wang et al., 2022). Spinal sensory neurons are involved in mediating the effects of various metabolic hormones and neurotransmitters, further highlighting their importance in regulating energy balance and physical activity. When in an energy deficiency state, hormones such as leptin cue the body to alter locomotor activity and thermogenesis (Affinati et al., 2023). It has also been suggested that leptin potentially communicates with sensory nerves in adipose tissue via DRG neurons activating a sympathetic reflex to induce BAT thermogenesis (Blaszkiewicz et al., 2019). Consistently, the observed metabolic disturbances were directly linked to the disrupted brain-adipose communication where bilateral denervation of interscapular (Hara et al., 2001) resulted in markedly decreased thermogenesis as well as increased body fat mass alongside reduced energy expenditure and “whitening” of the tissue (Blaszkiewicz et al., 2019). DRG afferents in WAT and BAT have also been implicated in regulation of adipose tissue mass, lipolysis, and thermogenesis. It was demonstrated that selective afferent denervation of epididymal WAT mimicked the compensatory increases in inguinal WAT observed previously with lipectomy. Based on these observations, they suggested that information related to lipid stores in different fat pads is conveyed via DRG to the brain to orchestrate compensation (Shi and Bartness, 2005). Follow-up experiments suggested that spinal afferents sense the level of lipolysis within visceral and subcutaneous WAT and change BAT thermogenesis via a central neural pathway involving the medulla, midbrain/pons, and hypothalamus (Song et al., 2009; Garretson et al., 2016; Ryu et al., 2017). Notably, mice with a whole-body deletion of sensory neurons expressing the transient receptor potential-v1 (TRPV1) ion channel exhibit lower core body temperature and reduced energy, while those lacking the calcitonin gene-related peptide (CGRP) exhibited an increase in basal metabolic rate and showed resistance to diet-induced obesity, indicating a potential role for these neurons in energy homeostasis (Walker et al., 2010; Garami et al., 2011; Makwana et al., 2021). Moreover, one study showed that fat thermogenesis and splanchnic-sympathetic nerve activity were significantly augmented in diet-induced obese mice post Roux-en-Y gastric bypass and that selective splanchnic denervation eliminated these energy findings that were induced by surgery (Ye et al., 2020). A more recent study, using a highly selective sensory ablation, confirmed that DRG neurons in WAT enhance lipogenesis and energy dissipation, suggesting that these sensory fibers can modulate sympathetic-mediated adipocyte thermogenesis (Wang et al., 2022). These findings further highlight the significant role of afferent fibers within adipose tissue in regulating energy expenditure and fat turnover and open avenues for future research on sensory innervation in interoceptive systems regulating variable metabolic functions.

Adipose tissue is exclusively innervated by sympathetic and sensory nerves, with no involvement from the parasympathetic system. While the role of sympathetic activation in promoting lipolysis is well established, increasing focus is being directed toward understanding the contribution of sensory nerves to adipose tissue. Sensory neurons play a crucial role in regulating thermogenesis in BAT, independent of sympathetic activity. For instance, injecting BAT with CGRP, a neuropeptide mainly released from sensory neurons, induced BAT thermogenesis (Osaka et al., 1998; Mishra and Townsend, 2023). Also, sensory denervation of WAT using capsaicin was found to suppress lipolysis and lead to adipose tissue hypertrophy (Shi and Bartness, 2005; Mishra and Townsend, 2024). Furthermore, recent findings indicate that lipolysis in inguinal WAT stimulates local sensory afferents, setting off a neural pathway between WAT and BAT, which in turn triggers acute thermogenesis in BAT (Garretson et al., 2016; Nguyen et al., 2017). Based on these findings, sensory afferents play a significant and impactful role in regulating adipose tissue and lipolysis.

## Molecular signaling pathways within spinal sensory neurons that regulate metabolism

Metabolism is a complex process comprising myriad biochemical reactions that oversee energy balance, maintain homeostasis and fuel cellular activities. Numerous factors regulate it, many involving a complex interplay between the gut and CNS. Spinal afferent neurons mediate a key role in transmitting signals from peripheral tissues—such as the adipose tissue, gut, and liver—to the CNS. In doing so, they relay vital information about nutrient availability and energy status to the brain, thus exerting a critical role in metabolism regulation (Münzberg et al., 2023). Multiple molecular and signaling pathways within spinal afferent neurons contribute to this communication, and by deciphering these pathways novel therapeutic targets may be uncovered for the management of metabolic disorders, such as obesity and type 2 diabetes, potentially revolutionizing the field of nutrition and metabolism. One pathway of interest involves the activation of the transient receptor potential (TRP) ion channels, specifically transient receptor potential vanilloid 1 (TRPV1) and transient receptor potential ankyrin 1 (TRPA1), in response to various gut-derived signals such as nutrients, gut hormones, and microbial metabolites. TRPV1 and TRPA1 are widely distributed throughout the body and are particularly prevalent in sensory neurons (Sanchez et al., 2001; Zygmunt and Högestätt, 2014; Borgmann et al., 2021; Maximiano et al., 2024). TRPV1 is notably abundant in abdominal sensory afferents and has long been investigated for its response to noxious stimuli (Hwang et al., 2005; Steiner et al., 2007). Mishra et al. revealed TRPV1 activation increases energy expenditure, decreases food intake, and protects against obesity, and plays a crucial role in promoting thermogenesis in BAT through Uncoupling Protein 1 (UCP-1) (Mishra and Townsend, 2023). Similarly, TRPA1 agonists improved metabolism and anti-obesity effects in rodents (Legrand et al., 2020). TRP activation in spinal afferents heightened energy expenditure and thermogenesis (Uchida et al., 2017; Legrand et al., 2020; Mishra and Townsend, 2023), along with an increased release of neuropeptides such as CGRP (Zygmunt et al., 1999) and substance P, which in turn transmit the energy signals to the brain via the spinal cord. CGRP is a neuropeptide expressed throughout the body and is heavily abundant in spinal sensory neurons and DRG (Noguchi et al., 1990; Lima et al., 2017). However, it presents a puzzling mechanism. Discrepancies arise between studies employing physiological genetic knockout and pharmacological exogenous manipulation. αCGRP knockout mice exhibit an increase in energy expenditure, thermogenesis, *β*-oxidation, and protection from diet-induced obesity with lower cholesterol levels and adiposity and improved glucose tolerance compared to CGRP wildtype counterparts (Walker et al., 2010; Lima et al., 2017). However, research on CGRP administration yields conflicting results. While one study reports lower food intake and total energy expenditure in CGRP-injected mice compared to controls (Sanford et al., 2019), other studies displayed that exogenous CGRP promotes appetite suppression, boosts energy expenditure through thermogenesis and lipolysis (Lutz et al., 1997; Lima et al., 2017; Sanford et al., 2019; Russo and Hay, 2023). These varied effects of CGRP on metabolism were previously shown to be modulated through sensory afferent neurons (Walker et al., 2010; Makwana et al., 2021). Endogenous GLP-1 acts locally on ileal enteric neurons instead of circulating as a hormone, while myenteric intestine-fugal neurons mediate its gastric and anorectic effects. Gastric neurons producing nitric oxide regulate appetite and gastric distension, with hypothalamic neurons detecting gastric volume via spinal afferent pathways (Zhang et al., 2022). A newly released paper showed that post-ingestion increases in sympathetic signaling to the gut suppresses L-cell specific release of GLP-1, suggesting a role of this gut hormone in post-meal energy and glucose regulation (Ren et al., 2024). Furthermore, emerging research highlights the impact of gut microbiota on the activity of spinal sensory neurons. Through the release of metabolites and microbial products, microbiota can modulate neuronal excitability and neurotransmitter release (Goswami et al., 2018; Le Roy et al., 2022; Dohnalová et al., 2022). This underscores the growing recognition of microbiota’s role in metabolism and energy regulation through the gut-brain axis (Van Hul and Cani, 2023).

A pivotal connection between microbiome metabolites and the brain occurs via sensory and vagal afferents (Dohnalová et al., 2022; Mayer et al., 2022; Cao et al., 2024), with TRPV1 neurons potentially meditating this signaling. Goswami et al. state that short-chain fatty acids (SCFAs) such as acetate, butyrate, and propionate decrease food consumption through sensory neuron activation, particularly vagal afferents (Goswami et al., 2018). Given the previously mentioned role of leptin in the regulation of sensory neuron activity in adipose tissue to promote sympathetic-mediated fat thermogenesis in a reflex mechanism (Shi et al., 2020) it would be interesting to explore if GLP-1 exerts a similar role through spinal sensory neurons. Adding to the complexity of this regulatory process is the finding from a previous study showing that the sensitivity of the intestinal GLP-1 action as a glucose regulator is impaired by gut microbiota dysbiosis in a rodent model of type 2 diabetes (Grasset et al., 2017).

Regarding receptors found on spinal afferents, cannabinoid receptor 1 (CB1) activation has been implicated in the regulation of food intake and energy balance as it has been shown to have orexigenic effects, leading to a transient increase in food intake but interestingly minimal body weight changes (Burdyga et al., 2010; Ye et al., 2020; Dohnalová et al., 2022). The negative or minimal effect of CB1 agonism on weight may be due to its positive effect in promoting physical activity (Dohnalová et al., 2022). This study showed that fatty acid amides stimulate CB1 expressing sensory neurons which in turn downregulate Monoamine Oxidase expression within the striatum leading to a surge in dopamine levels and a heightened drive to increase physical activity. Furthermore, CB1 knockout mice were shown to have reduction in food consumption, lower body weight and resistance to diet-induced obesity (Ruiz de Azua et al., 2021). Administration of CB1 inverse agonist to diet-induced obese mice results in significant weight loss by reducing food intake for a transient period, increasing energy expenditure and fat thermogenesis as well as splanchnic nerve activity. Interestingly, selective splanchnic denervation still caused weight loss; however, the anorectic effect of CB1 antagonism was eliminated (Ye et al., 2020). Another study showed that vagus-mediated CB1 signaling through peripheral CB1 regulate food reward and feeding behavior (Berland et al., 2022). Interestingly, a previous study using a transgenic mouse model of CB1 deletion in Phox2b-specific vagal neurons showed no effect of peripheral CB-1 in vagal afferents on body weight or food intake (Vianna et al., 2012). This suggests that peripheral CB1 signaling may be involved in food intake regulation, possibly through the afferent branch of the splanchnic nerve (i.e., spinal sensory neurons). AgRP is a neuropeptide primarily expressed in the AgRP/NPY neurons of the arcuate nucleus of the hypothalamus and has been robustly associated with promoting feeding behavior, increasing food intake, and body weight (Luquet et al., 2005; Aponte et al., 2011). AgRP overexpression in the hypothalamus causes significant hyperphagia and obesity (Ilnytska and Argyropoulos, 2008). Intestinal and portal glucose sensors signal spinal afferents to inhibit AgRP activity in the brain (Goldstein et al., 2021). Overall, the intricate network of molecular and signaling pathways within spinal sensory neurons, and their interplay, form a complex system expediting the communication of energy signals between the gut and the brain, highlighting their pivotal role in the regulation of energy balance and homeostasis (Williams et al., 2016). Furthermore, GLP-1 -a gut hormone secreted and released in response to food intake- plays an important role in glucose regulation and appetite control as it increases insulin secretion from the pancreas after eating and slows gastric emptying which can help with appetite control and prolong the feeling of fullness (Holst, 2007; Drucker, 2018). GLP-1-expressing myenteric neurons communicate with the CNS through afferent sensory neurons in response to post-ingestive gastric volume, which regulates meal termination and, sequentially, food intake (directly) and post-prandial glycemia (indirectly)(Zhang et al., 2022). Sensory nerves play a critical role in the insulinotropic action of GLP-1, particularly at lower doses. Capsaicin treatment, which deactivates sensory nerves, diminishes GLP-1’s ability to enhance insulin secretion at low doses, suggesting that at least part of the GLP-1’s incretin effect is mediated by DRG sensory neurons (Ahrén, 2004). In addition, portal vein afferent neurons that express CGRP were found to be essential for detecting low blood sugar levels and activating reactive glycemic response accordingly (Fujita et al., 2007). Selective elimination of TRPV1-expressing DRG neurons of the pancreas by capsaicin enhances insulin secretion in response to glucose stimulation and reduces post-absorptive blood glucose levels (Bou Karam et al., 2018).

**Figure 2:**
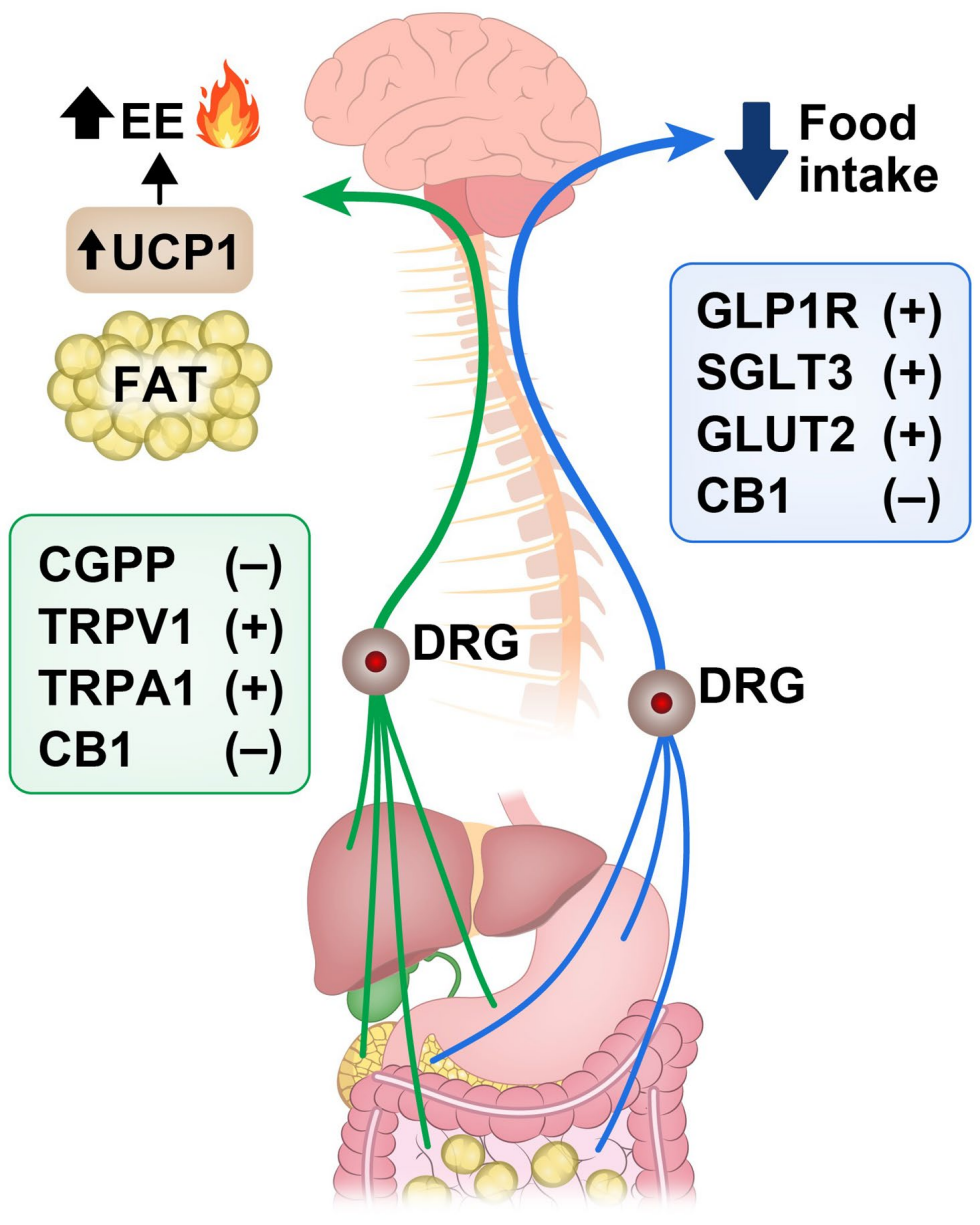
Signaling pathways in spinal sensory neurons that promote (+) or inhibit (−) reduction of food intake and increase in energy expenditure (EE) and fat thermogenesis along the gut-brain axis. Calcitonin gene-related peptide (CGRP); transient receptor potential vanilloid 1 (TRPV1); transient receptor potential ankyrin 1 (TRPA1); cannabinoid receptor-1 (CB1); Glucagon-like peptide-1 receptor (GLP-1R); sodium-glucose cotransporters type 3 (SGLT3); glucose transporters type 2 (GLUT2); uncoupling protein 1 (UCP1); dorsal root ganglion (DRG).

Molecular signaling pathways within spinal sensory neurons are pivotal in regulating energy balance and metabolism. These neurons serve as vital messengers, transmitting signals from peripheral tissues—like adipose tissue, gut, and liver—to CNS, providing crucial insights into nutrient availability and energy status. Among these pathways, activation of TRP ion channels, particularly TRPV1 and TRPA1, by gut-derived signals such as nutrients and microbial metabolites, sparks heightened energy expenditure and thermogenesis. This cascade also unleashes neuropeptides like CGRP and substance P, each playing a crucial role in this metabolic symphony. CGRP, abundant in spinal sensory neurons and dorsal root ganglia, exerts a dual influence by curbing appetite and modulating energy expenditure through intricate mechanisms. Gastrointestinal spinal sensory neurons were also shown to regulate food intake behavior by inhibiting hypothalamic AgRP neurons. In addition, peripheral CB1 signaling seems to be involved in regulating homeostatic food intake through spinal afferent pathways while CB1 modulation through vagal afferent pathways seems to regulate hedonic food intake as well as gut motility. Finally, the impact of SCFAs -secreted by gut microbiota on energy balance adds another layer of complexity, fine-tuning neuronal activity via spinal and vagal afferent pathways (Figure 2). These revelations underscore the dynamic role of spinal sensory neurons in maintaining energy balance and position them as promising targets for therapeutic interventions in metabolic disorders as we will discuss next.

## Potential therapeutics for targeting spinal afferent neurons

Several molecular and signaling pathways within spinal afferent neurons have been identified as key contributors in regulating energy balance. Therefore, targeting spinal afferent neurons for weight management has the potential to revolutionize the treatment of obesity and associated metabolic disorders. The complex signaling pathways within these neurons provide a promising avenue for the development of novel therapeutic interventions, as modulating the activity of spinal afferent neurons could lead to precise control of food intake, appetite suppression, and enhancement of energy expenditure. Since CGRP has shown distinctive impact on metabolism, there’s growing interest in exploring it as a potential therapeutic for obesity (Walker et al., 2010). Nilsson et al. found that αCGRP analog led to increased energy expenditure, decreased food intake, weight loss, and increased GLP-1 secretion in rodents (Nilsson et al., 2016). However, current studies lack conclusive evidence regarding its precise mechanism of action so using it as a therapeutic agent for obesity is still undergoing studies (Sonne et al., 2021). CB1 inverse agonist rimonabant was previously used in treating obesity, but due to its concerning negative impact on mood, it has been withdrawn from clinical use (Sam et al., 2011). Nevertheless, its significant influence on metabolism cannot be overlooked. Ye et al. demonstrated that CB1 inverse agonist mimics gastric bypass effects on weight loss and energy expenditure (Ye et al., 2020). Further research should focus on developing new methods to mitigate its side effects while retaining its anti-obesity properties. Recent Studies are speculating TRPV1 activation, and gut microbiota modulation for the treatment of metabolic diseases (Mishra and Townsend, 2023; Cani et al., 2019; Le Roy et al., 2022). Furthermore, GLP-1 receptor (GLP-1R) agonists are widely used for treating Type 2 diabetes due to their ability to enhance glucose-dependent insulin secretion, reduce glucagon levels, and minimize the risk of hypoglycemia (Drucker, 2018). Beyond glycemic control, these drugs also promote weight loss by reducing food intake, which has led to their approval for obesity treatment (Campbell and Drucker, 2013). Additionally, GLP-1R agonists have shown benefits for reducing cardiovascular mortality in non-diabetic obese population (Lincoff et al., 2023), and stop the progression of liver and neurodegenerative diseases such as Parkinson’s and Alzheimer’s (Drucker, 2018; Mahapatra et al., 2022).

## Conclusions and future directions of sensory neuron-driven obesity research

The influence of interoception on the brain’s decisions regarding energy balance hinges on humoral and neural communication between peripheral tissues and the brain. Neuronal pathways offer rapid and spatially precise communication, with sensory information reaching the brain via afferent nerves and motor commands being transmitted via efferent nerves. Conversely, humoral communication through circulating hormones and macromolecules provides slower, longer-lasting signaling that can reach multiple organs and serve various metabolic functions simultaneously. Vagal afferent neurons from the mesenteric gut play a well-recognized role in energy regulation, whereas the role of spinal afferents is less explored. Unlike vagal afferents, DRG or spinal afferents innervate a broader range of tissues, including subcutaneous adipose tissue, skeletal muscle, and skin, providing comprehensive sensory data crucial for energy regulation. The interaction between subcutaneous adipose tissue and the sympathetic nervous system in energy homeostasis is well-documented. However, the roles of skeletal muscle and skin in this context remain under-researched. Given their significant metabolic activity and roles in temperature regulation, these tissues could influence energy balance. Investigating the communication pathways between muscle, skin, and other organs, including the brain, is essential. While humoral signals such as myokines and hormones are part of this communication, they lack the speed and precision of neural signals. The complexity of spinal afferent circuitry has contributed to the limited focus on its role in energy homeostasis. Traditional surgical and chemical methods, which affect broad innervation and other peripheral nervous system pathways, have often produced misleading results. Modern neurobiological techniques are needed to dissect the functional specificity of DRG afferents and sympathetic pathways. Such advancements could enable targeted neuromodulation strategies to treat metabolic diseases and related conditions. The National Institute of Health (NIH) initiated the Stimulation of Peripheral Activity to Relieve Conditions (SPARC) program, given the relatively easy accessibility of the peripheral nervous system to manipulation and significant advancements using biomedical electronics and nanotechnology by the medical device industry (National Institutes of Health, 2024). V-Bloc® (Vagal Nerve Blocking Therapy), an FDA-approved device for the treatment of obesity, targets vagal afferent neurons to modulate signals between the stomach and brain, reducing feelings of hunger and promoting weight loss (Shikora et al., 2013). Future research might explore the use of other vagal afferent neuron manipulation techniques to mimic gut-brain pathways that can regulate mood and appetite, potentially aiding in curbing excess caloric intake.

On the other hand, spinal afferent neurons, which integrate visceral sensory information, contributing to the unconscious reinforcement of eating behaviors may be explored or manipulated to refine eating behavior therapies, providing more nuanced approaches to managing obesity by altering internal reward signals. Another promising avenue is investigating the gut microbiome’s role in modulating the gut-brain reward system, providing a mechanistic link between microbiome metabolism and brain reward circuitry. Behavioral therapies targeting implicit, low-road neural pathways may be more effective than those focusing on explicit factors, with the potential for neural reinforcement techniques to reduce physiological responses to food cues.
